# A framework for identification of on- and off-target transcriptional responses to drug treatment

**DOI:** 10.1038/s41598-019-54180-4

**Published:** 2019-11-26

**Authors:** Yi Huang, Masaaki Furuno, Takahiro Arakawa, Satoshi Takizawa, Michiel de Hoon, Harukazu Suzuki, Erik Arner

**Affiliations:** RIKEN Center for Integrative Medical Sciences, Yokohama, Kanagawa 230-0045 Japan

**Keywords:** Bioinformatics, Transcriptomics

## Abstract

Owing to safety concerns or insufficient efficacy, few drug candidates are approved for marketing. Drugs already on the market may be withdrawn due to adverse effects (AEs) discovered after market introduction. Comprehensively investigating the on-/off-target effects of drugs can help expose AEs during the drug development process. We have developed an integrative framework for systematic identification of on-/off-target pathways and elucidation of the underlying regulatory mechanisms, by combining promoter expression profiling after drug treatment with gene perturbation of the primary drug target. Expression profiles from statin-treated cells and HMG-CoA reductase knockdowns were analyzed using the framework, allowing for identification of not only reported adverse effects but also novel candidates of off-target effects from statin treatment, including key regulatory elements of on- and off-targets. Our findings may provide new insights for finding new usages or potential side effects of drug treatment.

## Introduction

The process of drug development is costly and time-consuming^1,2^. From pre-clinical tests to clinical trials, studies of new drug candidates assess both efficacy and safety, and comprehensively screen for adverse effects^3^. In the pre-clinical testing stage, the pharmacokinetics and pharmacodynamics of drugs are characterized by *in vitro* and *in vivo* studies, including absorption, distribution, metabolism, excretion, and persistence of pharmacological effects. In addition, drug safety is evaluated for unexpected and toxic effects on target tissues, carcinogenicity and mutagenicity. Once the pre-clinical tests ensure that the drug consistently produces the desired effects, the safety and dosing of the drug is determined through testing in cultured cells, animal models and healthy human volunteers in clinical phase I trials. In clinical phase II and III trials, the efficacy and safety of the drug are assessed on small and large cohorts of patients having the targeted disease. Post-marketing surveillance, known as phase IV, monitors adverse effects from long-term usage^4^. Several examples of adverse effects leading to withdrawal of drugs from the market have been reported. For example, Dextropropoxyphene that was patented in 1955 and used for analgesia, was withdrawn in recent years because of increasing risk of heart attacks and stroke^5^. Early detection of adverse effects (AEs) to ensure drug safety is important to prevent the harming of patients and to reduce the cost of drug development.

Many efficacious drugs have off-target effects, for example multi-kinase inhibitors for cancer therapies, and the off-target effect may cause adverse effects^6,7^. The potential to detect AEs in pre-clinical tests or clinical trials is limited by the number of participating patients, the duration of the studies and heterogeneity of populations^8^. In phase IV trials, post-marketing surveillance to monitor adverse events in real time is also challenging due to the passiveness of pharmacovigilance (drug safety) methods for collecting voluntary submissions through spontaneous reporting systems (SRSs) or mandatory submissions from healthcare center or pharmaceutical companies. Based on data from SRSs, several data-mining-based pharmacovigilance algorithms have been developed to perform disproportionality analyses to discover unexpected and adverse effects of drugs^9^. The results from these algorithms may be biased depending on the source of data, sampling variance and reporting bias. Even the multi-item gamma Poisson shrinker (MGPS) method that corrects data source bias has issues with high rates of false positives and negatives yet to be solved^10^. Recently, network-based methods have been developed that integrate chemical data with biological data sources for construction of AE networks, identifying putative mechanisms of AEs^11^.

Since the established algorithms for predicting AEs rely on reported data, developing an approach that can elucidate on- and off-target effects at the pre-clinical stage could allow for early identification of potential AEs, thus reducing the cost and time for drug development. In addition, comprehensive prediction of on- and off-target effects may be useful for drug repurposing, where new indications for existing drugs are identified. Drug repurposing has an advantage over the development of novel drugs, in that tedious and costly processes of drug development, especially for the safety concerns, may be bypassed. In 2006, Lamb *et al*. established the first database, Connectivity Map (CMAP) collecting gene-expression profiles from cultured human cells treated with 1,300 bioactive small molecules and providing interfaces for pattern-matching to mine these data. Using CMAP, there are several studies indicating novel usages for previously developed drugs^12^.

In this study, we establish an integrative framework to dissect on-/off-target effects of drug treatment at the molecular level, to help future identification of potential adverse effects. Using Cap Analysis of Gene Expression (CAGE) as the profiling technology, we measure the transcriptome activity at promoters in a quantitative and qualitative way, allowing for identification of the promoters/genes affected by the treatment, as well as high resolution identification of the regulatory elements involved in the regulation of the observed changes. We selected statins, commonly used for lowering cholesterol levels, as an example to evaluate our framework. Statins, inhibitors of 3-hydroxy-3-methylglutaryl-coenzyme A (HMG-CoA) reductase, have been majorly used for anti-hypercholesterolemia^13^, and may also influence intracellular signaling pathways and immune processes^14^. Several studies have suggested that statins may have potential as cancer therapy in human malignancies, including liver cancer^15,16^, breast cancer^17,18^, and leukemia^19,20^. In contrast, some studies revealed that statins increase cancer risk of prostate cancer^21,22^. In addition, there are other adverse effects of statins that have been identified in recent years, for example, increased risk of early-onset of type II diabetes^23^. However, the details of the mechanisms of these unexpected effects, whether positive or negative, are still not clear. Applying our framework to treatment of three different cell types with four widely used statins, we identify significantly associated pairs of transcriptional factors and pathways, and infer regulatory networks to elucidate the regulation of on-/off-target pathways. We show that our framework provides new insights about the effect of the drugs under study, and helps identification of new usages or potential side effects.

## Results

### Identification of on-/off-target effects of statins by transcriptome analysis

**Figure 1:**
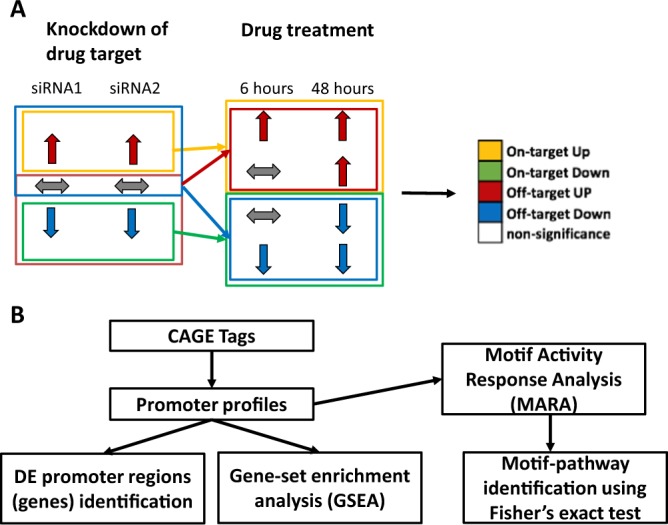
General framework and analytic pipeline. (**A**) Combining the expression profiles from statin-treated cells and HMGCR knockdown cells defines on-/off-target up-/down-regulated promoters or pathways. (**B**) CAGE tags are mapped to to FANTOM 5 pre-defined promoter regions, followed by identification of differentially expressed promoters or pathways. Subsequently, motif activity response analysis (MARA) is performed to identify differentially activated transcription factor binding motifs, and significantly associated motif-pathway pairs are identified using Fisher’s exact test.

The general framework is described in Fig. 1. We define on-target effects as the transcriptional events that result from inhibiting the main target of the drug under study using siRNAs, whereas off-target effects are defined as events after drug treatment that differ significantly from the on-target effects (Fig. 1A, up- and down-arrows indicate up- and down-regulation respectively). The analytical pipeline is outlined in Fig. 1B. CAGE tags are mapped to promoter regions and subjected to differential expression and gene set enrichment analysis, and Motif Activity Response Analysis (MARA) that associates observed expression changes to conserved transcription factor binding sites putatively regulating the response. On- and off-target effects may be cell-type specific, and also drug specific in the case that several drugs sharing the same main target are being investigated.

Using statins to evaluate the framework, CAGE experiments were performed to generate genome wide transcriptomic profiles from cells treated with four major statins on the market (Atorvastatin, Fluvastatin, Rosuvastatin and Simvastatin), as well as knockdowns of 3-hydroxy-3-methyl-glutaryl-coenzyme A (HMG- CoA), the main target of statin treatment. The human cultured cell lines selected were HepG2 (liver cancer cells), MCF-7 (breast cancer cells) and THP-1 cells (acute monocytic leukemia, AML, cells), which are related to statin-targeting organs or pleiotropic effects^14^. The 5′-capped RNA molecules are captured as CAGE tags, which reflect the transcription start sites and expression level of transcribed RNA molecules. The CAGE tags were mapped and annotated to the promoter regions identified in the FANTOM 5 project^24^ (Supplementary Fig. 1). Since HMG-CoA reductase (*HMGCR*) is the main target of statins, comparing the CAGE expression profiles between statin-treated and *HMGCR* knockdown cells allowed for investigation of putative on- and off-target effects of statins. We applied an ANOVA model to identify the differentially expressed promoters (DEPs) in statin-treated cells at two time-points (6 hours and 48 hours) after treatment of each statin. The DEPs were also identified in the two *HMGCR* knockdown experiments. Subsequently a step-wise filter was applied to define on- and off-target effects (Fig. 1). First, we filtered out the DEPs that showed inconsistent trends in the two knockdowns (using different siRNAs) of *HMGCR*. Second, for each individual statin, the DEPs with opposite trends after 6-hour treatment compared to 48-hour treatment were removed in order to focus on the long-term effects of statin treatment and to further filter out inconsistent candidates. Finally, we defined the remaining DEPs showing the same trend after *HMGCR* knockdowns as after statin treatment as on-target responders and the DEPs identified after statins-treated only, or reversely regulated compared to *HMGCR* knockdowns, as off-target responders (Table 1 and Supplementary Table 1). *HMGCR* itself was consistently observed to be upregulated after statin treatment in all cell types as previously reported^25,26^.

**Table 1:** Summary of differentially expressed transcripts in HMGCR knockdown and statin-treated cells.

	HepG2								MCF7								THP1							
	ato	atoOnly	flu	fluOnly	ros	rosOnly	sim	simOnly	ato	atoOnly	flu	fluOnly	ros	rosOnly	sim	simOnly	ato	atoOnly	flu	fluOnly	ros	rosOnly	sim	simOnly
*Off-targeting*																								
DN		162	1	2109		61		251	12	2936	13	3216	2	681	12	3162	13	7146	14	6473	9	5101	13	6665
UP	8	215	7	2258	7	119	6	416	42	2301	45	2648	10	711	39	2493	17	5432	18	4686	20	3262	18	4817
*On-targeting*																								
DN	2		9		1		5		40		40		15		42		9		5		1		2	
UP	7		11		1		9		36		43		45		55		1		1		1		2	

The number of on-/off-target promoters in different statin-treated cells. Abbreviations: Ato: Atrovastatin; Flu: Fluvastatin; Ros: Rosuvastatin; Sim: Simvastatin. AtoOnly: signficantly altered in Atrovastatin; FluOnly: signficantly altered in Fluvastatin; SimOnly: signficantly altered in Simvastatin. RosOnly: significantly altered in Rosuvastatin.

### Cell and statin specific responses at gene and pathway level

Surprisingly few promoters of genes could be consistently identified as common on-target responders, whether at the statin level or cell level **(**Supplementary Table 2). There were 16, 92 and 8 differentially expressed promoters commonly on-targeted by at least two statins in HepG2, MCF7 and THP1 cells respectively. The on-target responders of specific statin treatments were distinct in different cell lines, and only two on-target responders (*ACLY*, *MSMO1)* were found to be shared between HepG2 and MCF-7 cells (Supplementary Fig. 2). *ACLY* codes for the protein ATP citrate lyase which is the key enzyme responsible for Acetyl-CoA synthesis, in turn responsible for cholesterol biosynthesis. The cholesterol biosynthesis pathway has previously been reported to be upregulated by statin treatment^27^ and accordingly, the *ACLY* gene was found to be up-regulated in statin-treated cells in this study also. Similarly, methylsterol monooxygenase 1 (*MSMO1*), which was up-regulated in most of the statin-treated cells except for Rosuvastatin-treated HepG2 cells, is also involved in the cholesterol biosynthesis pathway. In addition to these two on-target responders, *FDFT1* was identified as an on-target responder common to MCF-7 and THP-1 cells. This gene encodes a membrane-associated enzyme involved in the mevalonate pathway in cholesterol biosynthesis. No up-regulated on-target responder common to HepG2 and THP-1 cells was identified and there were no down-regulated on-targets common to all three cell lines.

In contrast, most DEPs identified in statin-treated cells were off-target responders **(**Supplementary Tables 3 and 4). The number of shared off-target responders (shared in ≥2 statins) in each cell line were 462 up-regulated and 2,256 down-regulated promoters in HepG2 cells; 2,438 up-regulated and 4,365 down-regulated promoters in MCF-7 cells; and 5,156 up-regulated and 9,537 down-regulated promoters in THP-1 cells. However, only 37 and 124 up- and down-regulated promoters were common to all three cell lines. The commonly up-regulated genes were involved in the sphingolipid metabolism pathway, whereas the commonly down-regulated genes were enriched in several pathways, including systemic lupus erythematosus (SLE), alcoholism, shigellosis, and spliceosome (Supplementary Table 5).

The observation that few common on- or off-target responders could be identified in the three cell lines or across the four statins indicated that the molecular response may be cell-type dependent and statin specific at the gene level. We hypothesized that the response may be more similar at the systemic level, and proceeded with a gene sets based comparison approach in order to investigate the on- and off-target effects at the pathway level. Gene-set enrichment analysis (GSEA)^28^ was performed to assess the enrichment of 187 canonical KEGG pathways (12,875 genes covered) in the different conditions. The expression profiles in the three cell lines after *HMGCR*-knockdown or 6/48-hours statin treatment were analyzed by GSEA to identify significantly altered gene-signatures reflecting on- and off-target effects. Following the same rules identifying on- and off-target responders defined at the gene level, the on- and off-target pathways were identified based on the results of GSEA, by comparing gene-signatures enriched in statin-treated cells with signatures from *HMGCR* knockdown cells. The whole enrichment results are shown in Supplementary Fig. 3. There were 8, 10, and 12 significantly altered pathways partially on-targeted by one statin in HepG2, MCF-7 and THP-1 cells respectively, and 81, 80, and 115 significantly altered pathways partially off-targeted by one statin in HepG2, MCF-7 and THP-1 cells respectively. The numbers of on-/off-target pathways were similar among the three cell lines. However, the significantly altered pathways in HepG2 cells were generally more statin-specific than in MCF7 or THP1 cells.

**Figure 2:**
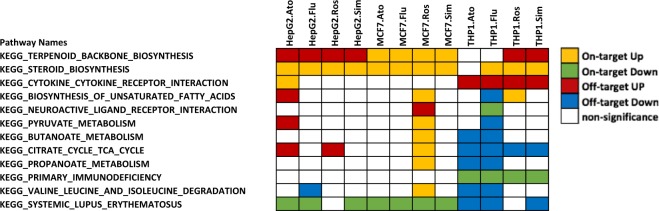
On-target canonical KEGG pathways in statin-treated cells. The matrix (pathways by samples) displays the on-target effects in at least one condition. Orange, green, red, blue and white indicate on-target up-regulated, on-target down-regulated, off-target up-regulated, off-target down-regulated and non-significantly altered pathways respectively. Abbreviations: Ato: Atrovastatin; Flu: Fluvastatin; Ros: Rosuvastatin; Sim:Simvastatin.

As expected, the on-target effects “Steroid biosynthesis” and “Terpenoid backbone biosynthesis pathway” were significantly up-regulated in *HMGCR* knockdown cells as well as in most statin-treatments (Fig. 2). This reflects a previously reported negative feedback up-regulation of steroid biosynthesis related genes after inhibition of HMG-CoA reductase activity in human lung tissues^29^. In addition, the “Systemic lupus erythematosus (SLE)” pathway was identified as on-target effect in HepG2 and MCF7 cells, but off-target effect in THP1 cells. There are several studies suggesting that statins could be used as SLE therapy, independent of cholesterol regulation^30–32^. In our GSEA results, the “Systemic lupus erythematosus (SLE)” pathway was significantly down-regulated in three cell lines after statin treatment (Fig. 2). Apart from the three pathways mentioned above, cell-independent on-target effects were not consistently detected in our experiments; most remaining enriched pathways were off-target effects, several of which have been previously reported. There are accumulating evidence suggesting therapeutic effects on human malignancies of statin treatment in multiple types of cancer cells, including MCF7 and THP1 cells. Supporting this notion, we observed that the “apoptosis” pathway was up-regulated and “cell cycle” was down-regulated after statin treatment in MCF7 and THP1 cells **(**Supplementary Fig. 3). Some rarely reported effects of statins such as reduction of proteasome pathways^33^, impairing the DNA repair mechanisms^34^ were identified as well **(**Supplementary Fig. 3).

### Identification of putative on- and off-target effects of statins

**Figure 3:**
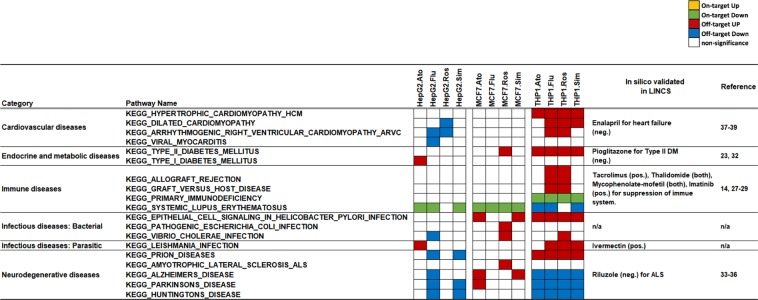
Off-target canonical KEGG pathways in statin-treated cells. The matrix (pathways by samples) displays the off-target effects in at least one condition. Orange, green, red, blue and white indicate on-target up-regulated, on-target down-regulated, off-target up-regulated, off-target down-regulated and non-significantly altered pathways respectively. Abbreviations: Ato: Atrovastatin; Flu: Fluvastatin; Ros: Rosuvastatin; Sim:Simvastatin. In silico validation and and whether the effect has been previously observed in the literature is also indicated.

In order to further examine our framework’s capability of identifying adverse or pleiotropic effects, we focused the analysis on disease pathways represented in KEGG. Out of 36 disease pathways identified to be on- or off-targets, 15 had various relations to cancer, containing a multitude of biological processes such as multiple signaling pathways, regulation of apoptosis, cell adhesion, in each pathway. We focused on the remaining 21 pathways, covering cardiovascular diseases, endocrine and metabolic diseases, immune diseases, infectious diseases, and neurodegenerative diseases. 19 of 21 pathways were significantly altered as on-/off-target effects of statins **(**Fig. 3**)**.

In each disease category, we could confirm at least one pathway through in silico validation (Methods) or literature support (Fig. 3 and Supplementary Table 6). One of the well-known adverse effects of statins is an increased risk factor of type II diabetes by statin treatment^23,35^. Accordingly, the “Type II diabetes mellitus” pathway was up-regulated as off-target effect in Rosuvastatin-treated MCF7 cells as well as in all statin treatments of THP1 cells. Furthermore, we found that statin treatment signatures were inversely correlated to an anti-type II DM drug, Pioglitazone, which is a type of thiazolidinedione medicine (TZD) that increases the sensitivity of insulin. The prevention of neuron degeneration diseases has been determined as off-target effects of statins in previous studies^36–38^; in line with this we detected several pathways related to neuron degeneration diseases down-regulated by statin treatment as well. In contrast, the pathway of amyotrophic lateral sclerosis (ALS) in Rosuvastatin-treated MCF7 cells was significantly up-regulated, lending support to previously shown associations between statins and ALS-like syndrome in clinical studies^39^ and further supported in silico by the observation that the signatures of Riluzole, which is used to treat amyotrophic lateral sclerosis (ALS), are inversely correlated to our statin-signatures in statins-treated cells. There are previous studies showing decreasing risks of heart failure from statin treatments by lowering cholesterol levels^40,41^, but there are also studies indicating that statin could cause heart disease^42^. The cardiovascular disease pathways were up-regulated in statin-treated THP1 cells as well. In addition, the signatures of drug used for heart disease, Enalapril, showed inverse correlation to several statin signatures.

Taken together, the results suggest that our approach could identify several previously reported adverse effects of statins, and provide putative novel adverse effects or novel indications of statins that may be followed up in future investigations.

### Identification of key regulatory elements of on-/off-target effects of statin treatment

In order to further elucidate the effects of statin treatment, we attempted to identify the key regulatory elements of the specific biological processes induced by statins. Taking advantage of the fact that CAGE pinpoints the exact locations of promotor activity which allows for precise identification of the cis-regulatory elements (motifs) implicated in regulating the promoter activity, Motif Activity Response Analysis (MARA)^43^ was used to identify the transcriptional regulatory dynamics associated with changes in expression after treatment with different statins in the three types of cells. In MARA, motif activities are modeled as a function of the number of transcription factor binding sites in the promotor regions and fitted to the expression levels of the promoters. After identifying the differentially activated motifs of transcriptional factors (TFs), we performed Fisher’s exact test to analyze the associations between activated motifs and GSEA-enriched pathways, based on the genes belonging to the pathways and the putative targets of each motif according to MARA. In total, 121,267 motif-pathway pairs were tested, with 778 pairs significantly (false discovery-rate < 0.05) associated. Analyzing each cell type separately, 278, 191 and 309 significant TF-pathway pairs (92 unique TFs) were identified in HepG2, MCF-7 and THP-1 cells respectively.

Based on our findings, the highly activated motifs of transcription factors *FOXD1/2*, *PATZ1*, and *TLX2*, were associated with the reported adverse effect, “Type II diabetes mellitus (Type II DM)” (Supplementary Fig. 4), a novel observation in this study. The most highly connected on-target pathway was “Systemic lupus erythematosus (SLE)”. Six unique motifs were associated to this pathway, including *E2F1..5*, *NR5A 1,2*, *POU2F1..3, TBP*, *TFDP1*, and *ZNF384*. The motif activities of these TFs were significantly (false-discovery rate < 0.05, ANOVA test) down-regulated and the results correspond to the down-regulation of “Systemic lupus erythematosus (SLE)” pathway in most of the statin-treated cells (Supplementary Fig. 5). *E2F1* has been previously suggested as a potential therapeutic target for SLE^44^. In addition, GWAS studies have indicated a risk allele of IL18 created by a variant in the binding sites of *POU2F1* (*OCT-1*) in SLE patients^45^. The over-expression of TBP has also been determined in SLE patients^46^. The down-regulation of the activity of these TFs suggest a potential mechanism for how statins suppress the SLE pathway. The other on-target pathway, “Steroid biosynthesis”, was associated with *KLF4* in MCF7 and THP-1 cells only.

We also identified four key motifs, *JUN*, *HIC1*, *NFKB1_REL_RELA*, and *TFAP2B*, putatively controlling programmed cell death of statin-treated cells as off-target effects (Supplementary Fig. 5). *JUN* and *NFKB1_REL_RELA* are well-known regulators of apoptosis, whereas *HIC1* have been reported as a hyper-methylated tumor suppressor gene in human malignancies^47^. The motifs of several transcription factors, including *E2F1..5*, *ELF1,2,4*, *ELK1,4_GABP{A,B1}, GFI1,NFY{A,B,C}*, *TFDP1* and *YY1*, were identified as putatively contributing to down-regulation of the “Cell cycle” pathway in MCF7 and THP1 cells (Supplementary Fig. 5).

## Discussion

In this study, we established an integrative framework that can elucidate on- and off-target effects of drugs, and identify putative regulators of these effects, by comparing expression profiles at the promoter level after drug treatment with the profiles obtained after gene perturbation of the primary drug target.

We used statins, which are conventionally used for cholesterol lowering and have several pleiotropic off-target effects, such as potential as anti-cancer drugs, to demonstrate utility of the framework. In order to characterize the regulatory mechanism of on/off target effects of statins, we used CAGE profiles to perform gene-set enrichment analysis (GSEA), and Motif Activity Response Analysis (MARA) to identify significant motif-pathway pairs contributing to the on- and off-target effects identified by comparing statin treatment and HMGCR (the primary target of statins) knockdown profiles. Off-target effects generally occur in any perturbation experiment. Therefore, we chose a conservative approach and created the on-target effective gene sets in this study by using negative control siRNA and two HMGCR-specific siRNAs, which minimizes the non-specific effects of siRNA treatment as well as the siRNA sequence-specific off-target effects. We observed that a very small amount of genes could be defined as on-target effect genes using these strict criteria. This suggests that HMGCR is an ideal drug target for cholesterol lowering, with minimum effect on other cellular functions.

For promoters of genes, we rarely identified common on- or off-target responders, neither across statins nor cell lines. Apart from a set of key genes in cholesterol biosynthesis, (ACLY, MSMO1 and FDFT1), there were no other differentially expressed promoters shared in at least two cell lines. In addition, we observed that statin treatment resulted in significant changes in a large amount of off-target genes. This is unlikely to be due to incomplete siRNA KD efficiency, since we rarely observed “off-target” genes common to all statin treatments. Thus, those off-target effects are likely to reflect differences in molecular structure of the four statins used here, where each statin may weakly associate with additional other cellular molecules or proteins. Because expression of those molecules may be variable between different cell-types, it is beneficial to use multiple cell-types for detection of off-target effects in this approach.

In contrast, there were many pathways shared among different statins and cell lines, such as the on-target effects up-regulation of “Steroid biosynthesis” and “Terpenoid backbone biosynthesis pathway” for negative feedback of statins, and off-target effects including the systemic lupus erythematosus (SLE) pathway which is down-regulated under statin treatment. In addition, the most well-known AE, the “Type II diabetes mellitus” pathway, was identified as an up-regulated off-target. Our finding suggests that statin treatment targets key pathways similar in different cell types rather than specific genes. Comparing our statin-induced gene-signatures with pre-compiled signatures from the LINCS database further validated our putatively identified on-/off-targets of statins. Signatures from drugs (RS-17053 and Niguldipine) that can decrease LDL and triglycerides and increase HDL were significantly enriched. In addition, several anti-cancer drugs signatures in the LINCS database were significantly associated as well. Furthermore, Type II DM therapies and PARP inhibitors showed inverse correlation with statin signatures. These results not only support our enrichment results but also show that our framework can dissect on- and off-target effects of drugs, and may be used for repositioning to other usages.

Although the framework can in principle be applied to any expression measurement technology such as RNA-seq or microarrays, the choice of CAGE allowed us to achieve a more accurate identification of regulators responsible for the on-/off-target effects using MARA. Our framework not only identified key regulators which have been previously reported for known off-target effects of statins, such as E2F1 and POU2F1 for systemic lupus erythematosus and JUN and NFKB1 for anti-cancer-related pathways, but also novel regulators (FOXD1/2, PATZ1, and TLX2) that may play important roles in statin-increased risk for Type II diabetes mellitus (T2DM). Some FOX (forkhead) transcription factors are associated with diabetes-related vascular disease^48^ where they have roles in the regulation of immune responses and inflammation. FoxD1 has been reported to play a role in the induction of plasminogen activator inhibitor-1 (PAI-1) to regulate the vascular function and modulate thrombosis, inflammation, and the extracellular matrix^48^. PATZ1 binds to DNA and plays an important role in chromatin modeling and since there are few genetic variants associated with T2DM (such as PPARG, KCNJ11 and TCF7L2), epigenetic regulation has been proposed to play significant role in T2DM instead^49^.

Using preclinical data can not only reduce the cost and time for drug development but also prevent patients from serious adverse effects (AEs) in the early stages of drug treatment. However, a method that systematically uses preclinical data to identify putative AEs is still lacking. The framework presented here may aid in prioritizing among candidates when some of them may have suggested AEs at the molecular level.

Based on a similar experimental design, our framework can be applied to other drugs in the future. For example, the pan histone deacytelatse (HDAC) inhibitors have been used as anti-cancer drugs or neurodegenerative disease^50–52^. There are three major classes of HDACs. The pan inhibitors inhibit the enzyme activity for all kinds of HDACs, whereas identifying class-specific HDAC inhibitors or dissecting the on- and off-target effects of specific classes of HDACs are still unmet clinical needs. Our study shows the capability of identifying unexpected effects including potential adverse effects by preclinical data from human cultured cell lines. Applying this framework in early stage of drug development may filter out the drug candidates with potential AEs to reduce time and costs.

## Methods

### Statin treatment, knock-down, cap analysis gene expression (CAGE) library preparation and sequencing

HepG2 and MCF-7 cells were cultured in DMEM high glucose (Gibco) supplemented with 10% inactivated fetal bovine serum (Gibco), 100 U/mL penicillin, 100 μg/mL streptomycin (Gibco) and 1 mM sodium pyruvate (Gibco). THP-1cells were cultured in RPMI-1640 (Gibco) supplemented with 10% inactivated fetal bovine serum (Gibco), 100 U/mL penicillin and 100 μg/mL streptomycin (Gibco). In the statin treatment, Atorvastatin, Fluvastatin, Rosuvastatin and Simvastatin were added to the culture medium at the final concentration of 30 μM for MCF-7 and THP-1 and 10 μM for HepG2 for 6 and 48 h. In the siRNA treatment, 2 siRNAs against *HMGCR* gene (HSS104864and HSS179267, Thermo Fisher SCIENTIFIC) and negative control siRNA (Stealth RNAi siRNA Negative Control, Med GC, Thermo Fisher SCIENTIFIC) were transfected using Lipofectamine RNAiMAX Transfection Reagent (Thermo Fisher Scientific) and incubated for 48 h. The *HMGCR* knockdown efficiency for HSS104864 and HSS179267 was 80% and 81% (HepG2), 42% and 52% (MCF-7) and 77% and 70% (THP-1), respectively. After the treatments, total RNA was extracted from the cells using RNeasy kit (Qiagen) and was subjected to the CAGE analysis using the nAnT-iCAGE protocol. Triplicate data was produced for statin and siRNA treatments, and 6 replicate data for control of the statin (1% DMSO at final concentration) and negative control siRNA treatments. CAGE libraries were sequenced on Illumina HiSeq. 2000. CAGE data is available at Gene Expression Ominbus (accession number GSE134817).

### CAGE data processing

We used the MOIRAI pipeline^53^ for processing CAGE tags generated. Briefly, bwa and delve was used for mapping with default parameters. We used the first position of the start of reads mapped on the positive strand, and the last position of the end of reads mapped on the negative strand, as the CAGE tag positon. CAGE tags were mapped to robust decomposition peak identification (DPI) clusters defined and annotated in FANTOM 5 database^24^ using the intersectBed function of bedtools^54^. The expression profile of each sample was merged to a DPI clusters-by-sample table as input for edgeR^55^. The read counts of tag-clusters were normalized by “relative log expression (RLE)” method and the normalized expression table was subsequently filtered to discard tag-clusters expressed at less than five counts per million (CPM) in all samples of each cell line. In addition, the voom function from limma^56^ was used to transform RLE-normalized CPM values to fit a normal distribution. The voom-transformed data was used for gene-set enrichment analysis (GSEA) and motif activity analysis. For GSEA, the CPM of tag-clusters belonging to the same gene annotated in the FANTOM 5 database was summarized to represent expression levels at the single gene level. Log2-transformed CPM profiles were applied to Partek® Genomics Suite® software, version 6.6 Copyright ©; 2016 Partek Inc., St. Louis, MO, USA. for batch-removal, identification of differentially expressed tag-clusters (DETs) by ANOVA, and data visualization. For identification of DETs a false discovery rate (q-value) threshold was used (≤5%).

### Gene-set enrichment analysis (GSEA)

GSEA aims to identify whether members of a gene-signature predefined by prior biological knowledge tend to be distributed at the top or bottom of a sorted gene list based on expression profiles associated to distinct phenotypes, such as case vs. control or treated vs. untreated^28^. An advantage of using GSEA for analyzing drug response is that not only strongly regulated genes, but also the trends of gene expression in pathways, are considered. The GSEA software was downloaded from the Broad Institute website and installed locally and (http://www.broad.mit.edu/gsea/). This algorithm uses a normalized Kolmogorov-Smirnov statistic to identify gene-signatures that are significantly associated with gene expression profiles of distinct phenotypes. An enrichment score (ES) of specific gene-signature are calculated for each phenotype; a normalized ES (NES), which is divided by the maximum ES in individual enrichment analysis, is used to compare the same gene-signature in different experiments. The biological knowledge base used in GSEA is the Molecular Signatures Database (MSigDB) v5.0. First we merged the expression values of promoters belonging to the same gene as the summarized value to represent the expression level of each gene. A ranked gene expression profile was generated based on the built-in signal-to-noise algorithm. GSEA identifies the most represented gene-patterns where the p-value is generated from permuting the members in a given gene-signature 1000 times. For the sensitivity and specificity of the enrichment analysis, the sizes of individual gene-signatures were limited from 5 to 500. Based on this filtering, 168 of 187 KEGG pathways remained for enrichment tests. The threshold of significance that was used followed the recommendation from GSEA manual, with p-value ≤ 0.05 or FDR ≤ 20%.

### In-silico validation

In order to assess the validity of identified on-/off-target pathways, we selected the top 50 and bottom 50 genes from each combination of statin and cell type, sorted by the statistical significance of their differential expression as signatures to compare with the 3,096 drug-induced signatures that could be queried by lincscloud (http://lincscloud.org) in the LINCS (Library of Network-Based Cellular Signatures) database. In order to verify the specificity of each signature from different cell lines treated with statins, 50 randomly selected up- and down-regulated gene-signatures from the human whole transcriptome were also generated 100 times to query the database for comparison of the results from random queries with the original signatures. As following the recommendation from lincscloud website, we used the mean connectivity score (score_best4) across the four cell lines in which the pertubagen connected most strongly to the querying signatures to represent the standard of similarity. Then, one-sample T-test was performed to examine the significance between the score of specific drug from our querying signature and random data-sets. In total, there are 3,096 drug-signatures in LINCS database that were compared. The signatures are defined as significantly enriched by score_best4 ≥ 80 or score_best4 ≤ −80 with FDR ≤ 5%.

### Identification of significantly associated motif-pathway pairs

In brief, according to the motif occurrence in the regions −300 to +100 base pairs (bp) from the peak of FANTOM 5 robust DPI clusters, MARA describes the ability of motifs to significantly explain the variance in expression between *HMGCR* knockdown and statin-treated cells. First, we calculated the binding profiles of 190 transcriptional factors (TFs) defined as central motifs by FANTOM 5 with respect to the transcriptional start site (TSS). Second, the putative binding sites of TFs were associated with FANTOM 5 promoter regions. The threshold on the number of predicted TFBSs for each motif defaults to 150. In final, the motif activities were calculated. This step will generate a single file containing the motif activities in each condition, their standard deviations, and their overall Z-scores. The ANOVA was performed to identify differentially activated motifs under 48-hours treated vs. control samples. Based on the information of putative targets on promoters, we performed Fisher’s exact test to examine the number of genes which belongs to the specific pathway, and identified cases where the stargeting by a specific TF was significantly higher than for the whole transcriptome background. There were 778 of 121,267 motif-pathway pairs significantly associated at false discovery-rate < 0.05).

## Supplementary information

Supplementary Info

Dataset 1
