# Supplementary material for: A framework for identification of on- and off-target transcriptional responses to drug treatment
Source: Sci Rep. 2019 Nov 26;9:17603. doi: 10.1038/s41598-019-54180-4 (PMC6879629; doi:10.1038/s41598-019-54180-4)
Supplement: Supplementary file 1 — Supplementary Info [file 41598_2019_54180_MOESM1_ESM.pdf]

Supplementary Information

A framework for identification of on- and off-target transcriptional responses to drug treatment

Yi Huang, Masaaki Furuno, Takahiro Arakawa, Satoshi Takizawa, Michiel de Hoon, Harukazu Suzuki, Erik Arner

Supplementary Figure 1. Mapping ratio of CAGE tags

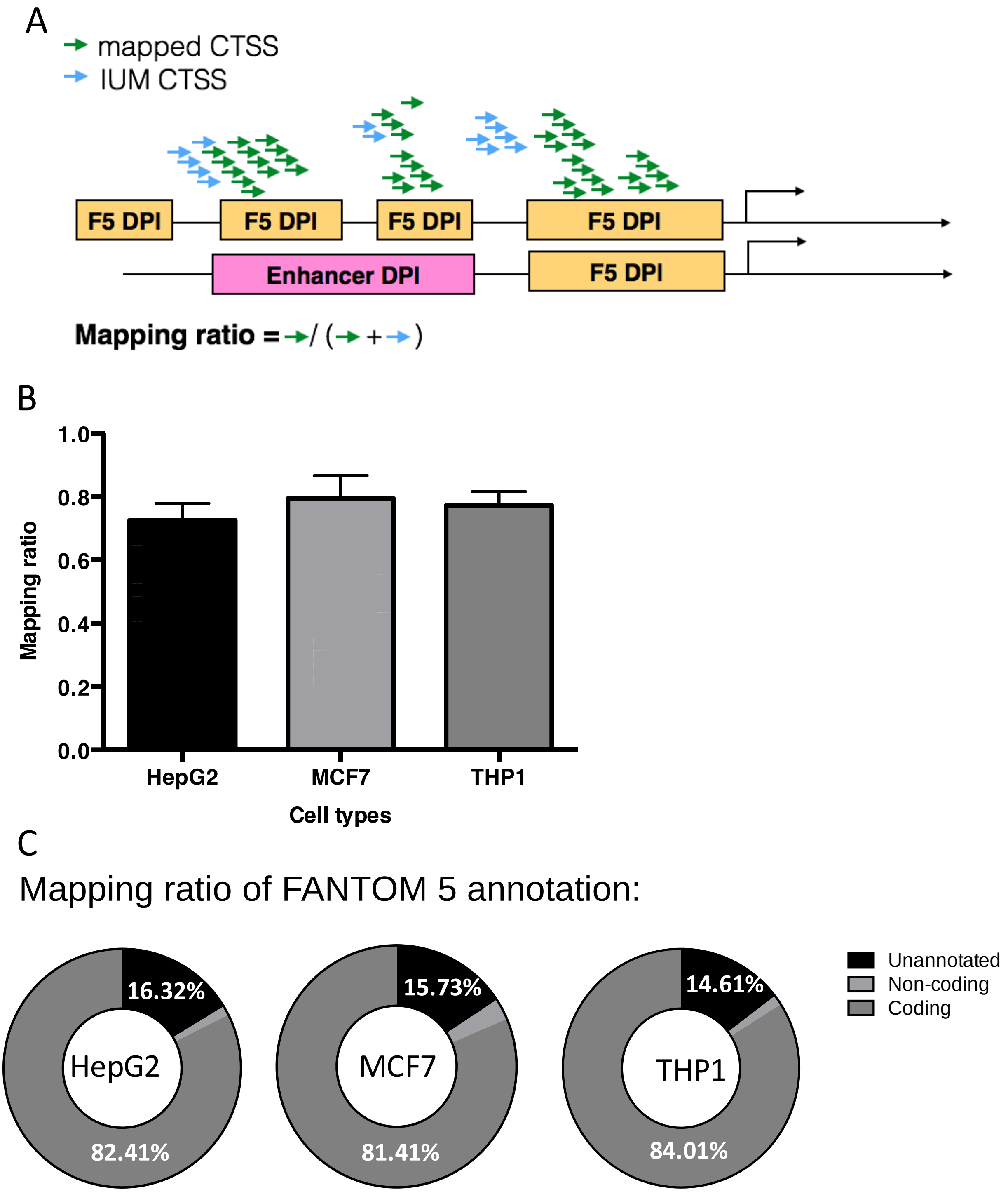

Supplementary Figure 1. **Mapping scheme of CAGE tags.** (A) Scheme of mapping CTSS to DPI clusters. CAGE tags were mapped to robust decomposition peak identification (DPI) clusters defined and annotated in FANTOM 5 database by intersectBed function of bedtools. The mapping ratio was calculated as shown in each cell type (B). The proportion of mapped regions are shown for each cell type in (C).

Supplementary Figure 2. Shared on-targets of statins in different cell types

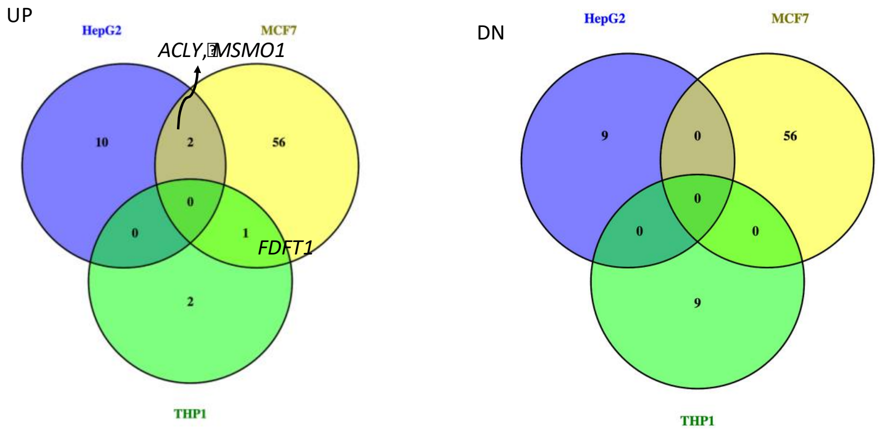

ACLY encodes for the protein ATP citrate lyase which is the key enzyme responsible for synthesis of acetyl-CoA synthesis
MSMO1 (Methylsterol monooxygenase 1) is involved in the cholesterol biosynthesis pathway.
FDFT1 encodes a membrane-associated enzyme involved in the mevalonate pathway in cholesterol biosynthesis

Supplementary figure 2. Overlap between statin treatments. The Venn diagrams show the overlap between statin treatment at the promoter levels between HepG2 (blue), MCF-7 (yellow) and THP-1 (green) cells. Left: up-regulated promoters; Right: down-regulated promoters.

Supplementary Figure 3. Summary of GSEA

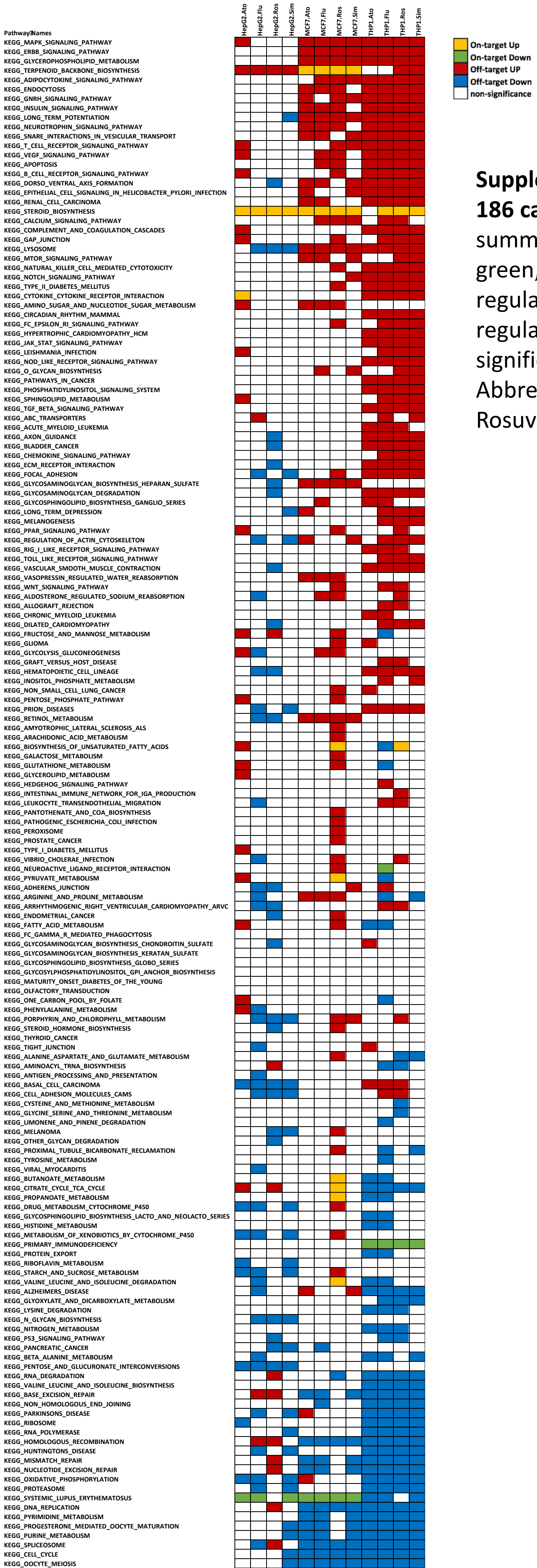

Supplementary Figure 3. **A summary of GSEA results of 186 cannoical KEGG pathways.** The GSEA results summarized as a pathways by samples matrix. Orange, green, red, blue and white indicate on-target up-regulated, on-target down-regulated, off-target up-regulated, off-target down-regulated and non-significantly altered pathways respectively. Abbreviations: Ato: Atrovastatin; Flu: Fluvastatin; Ros: Rosuvastatin; Sim:Simvastatin.

Supplementary Figure 4. Significantly activated motifs in Type II DM pathways

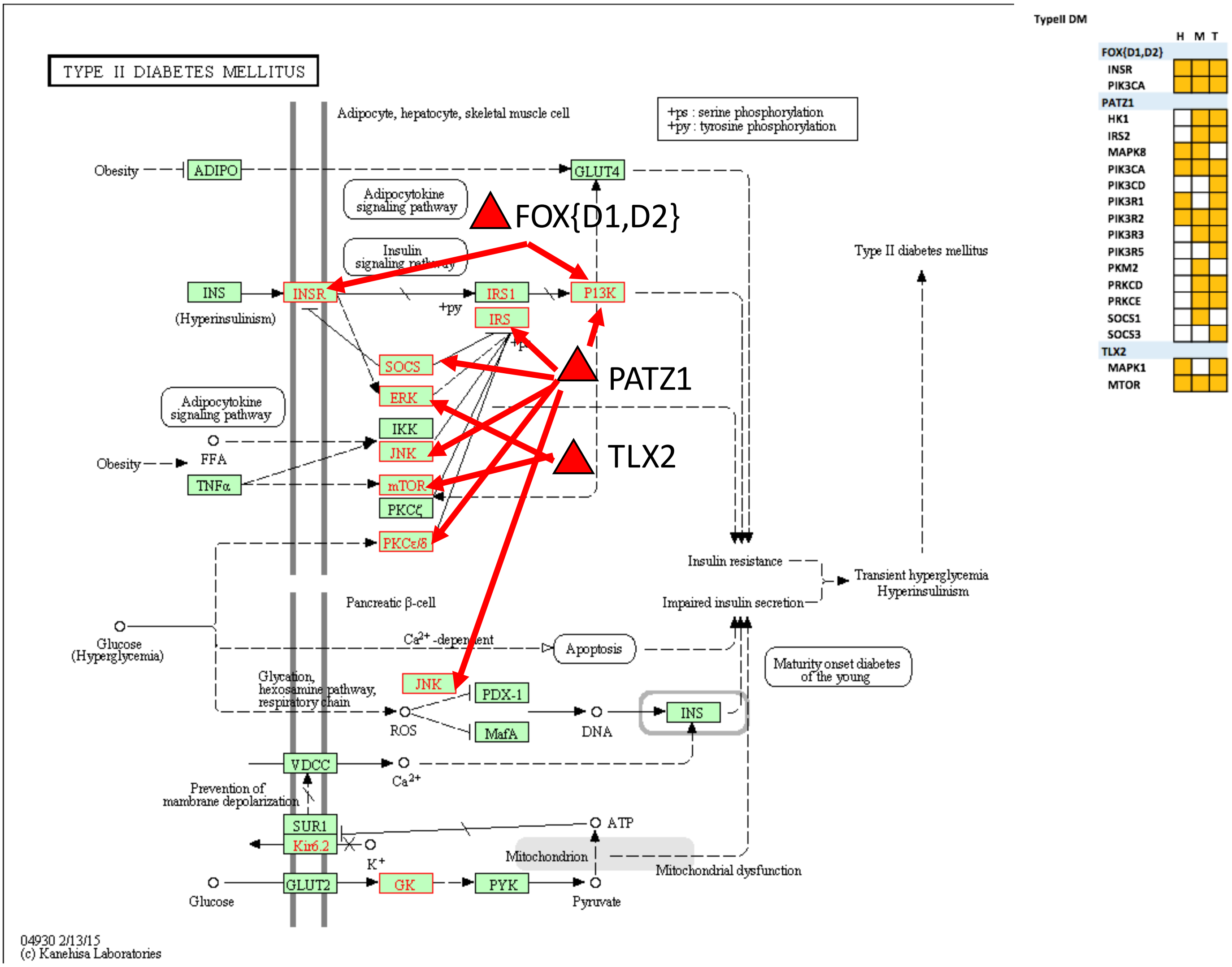

Supplementary figure 4. Significantly activated motifs in Type II DM pathways. Red triangles indicates the activated motifs in KEGG Type II DM pathway and red arrows indicates motifs and their putative targets. The left panel shows the activated motifs and putative targets in different cell lines. KEGG imagery modified from [Kanehisa, M. and Goto, S.; KEGG: Kyoto Encyclopedia of Genes and Genomes. *Nucleic Acids Res*. 28, 27-30 (2000)] and used with permission.

Supplementary Figure 5. Significantly associated motif-pathways by cell types

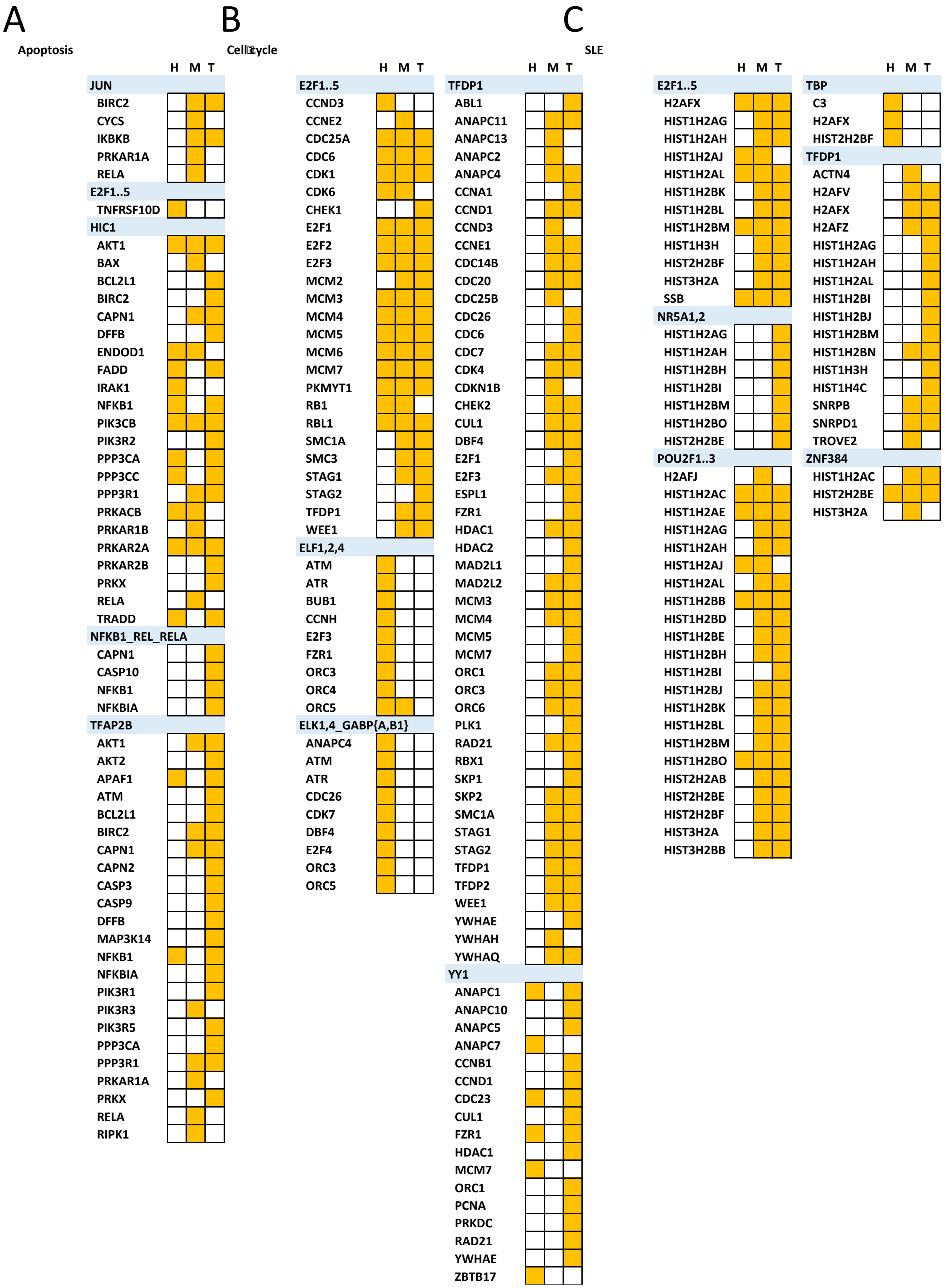

Supplementary figure 5. The significantly associated motif-pathways for (A) apoptosis, (B) cell cycle and (C) Systemic Lupus Erythematosus (SLE) are shown as heat-map like plots. Yellow blocks indicate the signficant association between motif and pathways in specific cell types. H: HepG2 cells; M: MCF7 cells; T: THP1 cells.
